# Crystallography Databases Hunt for Fraudulent Structures

**DOI:** 10.1021/acscentsci.3c01209

**Published:** 2023-10-09

**Authors:** Dalmeet Singh Chawla

In 2022, a study revealed that about 800 papers published in crystallography and exotic-chemistry journals originated from a paper mill.

**Figure:**
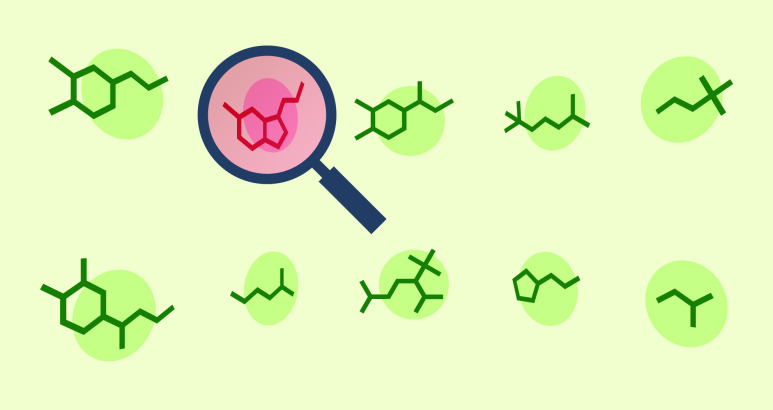
Credit: Shutterstock/Will Ludwig/C&EN.

At the time, popular scientific sleuth David Bimler noted in the study that the papers in question appeared to recycle images, cite irrelevant papers, and contain irregularities in their method sections.

Paper mills are known for churning out made-up, plagiarized, and subpar papers. When one of its articles is accepted by a journal, the paper mill sells authorship slots to academic scientists trying to artificially inflate their publication count.

In response to Bimler’s study, the Cambridge Structural Database (CSD), one of the most popular crystallography databases, flagged 992 crystal structures mentioned in the implicated papers. As of April 2023, the CSD had formally retracted 209 of the flagged structures after journals retracted some of the implicated papers. But many of the flagged structures remain in the database because they come from articles in predatory journals, which often have no interest in retracting papers they know to be fraudulent.

Databases have established systems for spotting fraud both when scientists submit a structure and when users later browse the database. There seems to be a consensus that databases such as the CSD are doing a good job of weeding out fraudulent crystal structures. Meanwhile, databases are recruiting experts to check the validity of structures and making it easy for users to review one another’s submissions.

The CSD is the largest repository of its kind, hosting about 1.25 million crystal structures and related data of small molecules and metal–organic compounds. Researchers can search structures in databases such as the CSD to identify promising new drug candidates and semiconductors, for example.

One unique aspect of crystallography data is that researchers can authenticate one another’s structures by processing the data contained in a crystallographic information file (CIF). Among other data, the CIF can include structure factors, which mathematically represent how the crystal diffracts X-rays and thus reveal where atoms are located in the crystal’s lattice. “That tends to be a very powerful thing within the crystallographic community because it’s very difficult to produce fraud that is impossible for another scientist to spot,” says Gregory Ferrence, a chemist at Illinois State University who has written about the CSD.

Each structure that is submitted to the CSD is automatically scanned with checkCIF, software created by the International Union of Crystallography (IUCr), which reports on the consistency and integrity of crystal structures. The software can identify crystal structures that have undergone manual changes, such as the positions or type of atoms in the structure. Submissions are also checked against all other existing structures in the database. Researchers typically commit this type of fraud by downloading, doctoring, and resubmitting CIF data.

Suzanna Ward, head of database and community at the Cambridge Crystallographic Data Centre (CCDC), a University of Cambridge body that manages the CSD, notes that validating crystallographic data is becoming more complicated.

For example, current checkCIF software was initially designed to validate single-crystal structures acquired using X-ray diffraction. But researchers now need to validate crystal structures of powders and structures coming from other techniques, such as electron diffraction. Future versions of checkCIF might be better able to handle these and other cases as new crystallography techniques arise, Ward says.

Another challenge databases such as the CSD must navigate when looking for fraud is that they currently rely on journals to retract papers before taking similar action on their end. As a result, such databases are introducing greater scrutiny of crystals by doing peer review in collaboration with journals, Ward tells C&EN. Steps taken include hiring more staff dedicated to policing research integrity issues and establishing new relevant policies, processes, and guidelines.

Under the CSD’s current process, when a paper goes to a journal for peer review, the CSD shares information about crystals with journal editors and peer reviewers, Ward says. Every single entry is checked by an in-house entry-level doctorate scientist and an expert in crystallography, she adds. They validate a structure’s novelty and plausibility by comparing it to past submissions and using software that predicts how molecules pack in crystals.

Journals usually require that structures in manuscripts include a CCDC deposition number—a code indexing a crystal structure in the CSD—which the author includes with the submission. Publishers can then access the prepublication data from the CCDC as well as checkCIF validation reports and send these to peer reviewers evaluating the paper. If the paper is eventually accepted and published, journal editors would notify the CCDC, which then would release the associated crystal structure and data publicly.

Despite checkCIF and this exchange between databases and journals, François-Xavier Coudert, a computational chemist at the French National Center for Scientific Research, says he doubts that the CCDC has spotted all the fraudulent structures in its database. “I think they caught the people who faked structures but not very cleverly,” he says. “I’m pretty sure there are ways of getting past the screening that they did.”

“A physically realistic, carefully designed fraudulent structure could probably not be caught,” Coudert says. CheckCIF and similar software have their limits, so a fraudulent file that is good enough could slip by, he adds. Still, it is not easy to produce such a fraudulent file, and Coudert doubts that thousands more fake structures linger in chemical databases.

Databases such as the CSD face a challenge to police themselves and weed out inferior and fake science, Ferrence says. Initially, he was bothered that the CSD was merely flagging the possibly fraudulent structures and not retracting them. But he changed his mind as he got to understand the roles of databases more. Their job “is to archive what has ... been peer-reviewed and accepted by various journals or other outlets,” Ferrence adds. “It is not generally the job of the CCDC to be the peer reviewer.”

Ferrence argues that it is unfair to expect peer reviewers to actively detect any fraud or misconduct in papers. If reviewers have to wonder if the work is real or fake, they cannot think as deeply about the science, he says. “I’m spending my intellectual peer review bandwidth looking for fraud and not doing what I’m supposed to be doing, which is evaluating the science on the merits of the science.”

Andrew Allen, a physicist at the U.S. National Institute of Standards and Technology and the editor in chief of IUCr journals, tells C&EN that his publications work closely with databases to make sure crystal structures have not been published before and to verify that structure factors have not been derived from previously published articles. They also carry out data validation checks on CIFs and structure factors and keep an eye out for new forms of fraud and manipulation.

Alex Stanley, CEO of the IUCr, says the organization has guidelines for journal publishers. The guidelines suggest that all papers submitted for review should include the coordinates of atoms and the structure factors of crystals. To generate a CSD deposition number to send to journal reviewers, the database requires both a CIF and a structure factor file, according to Stanley. But she says that despite the guidelines, journals are still publishing papers without the relevant structure factors, particularly journals that take less care with the data.

The CSD has been quite proactive in dealing with issues of fraud, Stanley says. She points out that in 2018 the repository hired a dedicated data integrity scientist, who focuses on making sure the CSD’s data coverage does not have gaps and who works closely with the IUCr to devise new methods of identifying fraud and plagiarism.

“Our data integrity scientist works with our team of scientific editors, scientists, and developers to look at a range of data integrity issues,” Ward says. The CSD does not provide much information on its methods so as not to aid would-be fraudsters, she adds.

Other repositories are taking different approaches. The Crystallography Open Database (COD) holds about half a million structures and has caught 159 instances of fraud since its inception in 2003. The COD plans to pilot a crowdsourced system for peer review using an interactive web application that volunteer crystallographers can use to check structures.

Saulius Gražulis of Vilnius University, who runs the COD, says that his former student Monika Kaltenytė built the application. He says the repository plans to roll out the system in the coming months.

According to Gražulis, the new software scans every submission to the COD and checks for a number of criteria, including the presence of publication metadata and consistent author names. The system automatically sends suspicious submissions for external peer review before they are included in the database.

“We will have to rely on the crystallographic community to do these reviews,” Gražulis says. “But we hope that having independent reviews from different experienced crystallographers would spot strange patterns, including fraud.”

## Dalmeet Singh Chawla is a freelance contributor to

Chemical & Engineering News, *the independent news outlet of the American Chemical Society.*

